# Midlife women, bone health, vegetables, herbs and fruit study. The Scarborough Fair study protocol

**DOI:** 10.1186/1471-2458-13-23

**Published:** 2013-01-10

**Authors:** Caroline A Gunn, Janet L Weber, Marlena C Kruger

**Affiliations:** 1 Institute of Food, Nutrition and Human Health, Massey University, Private Bag 11222, Palmerston North, 4442, New Zealand

**Keywords:** Bone, Osteoporosis, Postmenopausal, Fruit, Vegetables and herbs, Net endogenous acid production, Inflammation, Phytochemicals

## Abstract

**Background:**

Bone loss is accelerated in middle aged women but increased fruit/vegetable intake positively affects bone health by provision of micronutrients essential for bone formation, buffer precursors which reduce acid load and phytochemicals affecting inflammation and oxidative stress. Animal studies demonstrated bone resorption inhibiting properties of specific vegetables, fruit and herbs a decade ago.

Objective: To increase fruit/vegetable intake in post menopausal women to 9 servings/day using a food specific approach to significantly reduce dietary acid load and include specific vegetables, fruit and herbs with bone resorbing inhibiting properties to assess effect on bone turnover, metabolic and inflammatory markers.

**Methods/Design:**

The Scarborough Fair Study is a randomised active comparator controlled multi centre trial. It aimed to increase fruit and vegetable intake in 100 post menopausal women from ≤ 5 servings/day to ≥ 9 servings/day for 3 months. The women in the dietary intervention were randomly assigned to one of the two arms of the study. Both groups consumed ≥ 9 servings/day of fruit/vegetables and selected herbs but the diet of each group emphasised different fruit/vegetables/herbs with one group (B) selecting from a range of vegetables, fruit and culinary herbs with bone resorbing inhibiting properties. 50 women formed a negative control group (Group C usual diet).

Primary outcome variables were plasma bone markers assessed at baseline, 6 weeks and 12 weeks. Secondary outcome variables were plasma inflammation and metabolic markers and urinary electrolytes (calcium, magnesium, potassium and sodium) assessed at baseline and 12 weeks. Dietary intake and urine pH change also were outcome variables. The dietary change was calculated with 3 day diet diaries and a 24 hour recall. Intervention participants kept a twice weekly record of fruit, vegetable and herb intake and urine pH.

**Discussion:**

This study will provide information on midlife women’s bone health and how a dietary intervention increasing fruit and vegetable/herb intake affects bone, inflammatory and metabolic markers and urinary electrolyte excretion. It assesses changes in nutrient intake, estimated dietary acid load and sodium: potassium ratios. The study also explores whether specific fruit/vegetables and herbs with bone resorbing properties has an effect on bone markers.

**Trial registration:**

ACTRN 12611000763943

## Background

Osteoporosis meaning “porous bone” is the term for inadequate bone mass. It is a global problem seen most often in the elderly and in women (80%) [1] and is considered one of the ten most important diseases affecting the world’s population [2] and is particularly prevalent in developed countries with ageing populations and longer life spans [3]. Bone loss is accelerated at early menopause resulting in increasingly fragile bones prone to breakage. Inflammation also increases with age and exacerbates bone loss [4-6].

Osteoporosis poses a significant health and economic burden for New Zealand families and the public health system. The number of older (> 50 years) New Zealanders is increasing steadily and the cost of treating fractures and secondary illnesses related to osteoporosis is expected to rise from $330 million in 2007 to $458 million by 2020 [3].

Fruit and vegetables (F/V) are positively associated with bone status. The beneficial effect is thought to be through provision of micronutrients potassium, magnesium, calcium, vitamins A, C, E and K, and potentially a lower dietary acid load conferred by the fruit and vegetables food group [7-9]. Typical western diets are acidic because predominantly acid (hydrogen ions) rather than base (bicarbonate) is created during the metabolism of the daily food intake. Acid forming grains and high protein food derived from animal origin (meat, fish and eggs) contain sulphur based amino acids, methionine and cysteine which create acid when metabolized. Alkaline forming foods contain potassium salts which can be broken down to make alkaline buffers [10]. Vegetables and fruit are considered alkaline because of their high mineral content in the form of salts of organic acids. The salts, predominantly potassium based but also calcium and magnesium, generate bicarbonate to balance the acid produced from the rest of the diet.

Western diets are low in F/V and high in grains and animal protein compared to the typical diet of early man. The change from plant based diets to modern, western diets characterized by foods that are acid rather than alkaline forming results in a low grade systemic metabolic acidosis [11-13]. The level of acidity created can be estimated from the dietary intake. A significant change in estimated net endogenous acid production (est.NEAP) is said to have occurred from pre agricultural times (−88mEq/d) to today (+ 48 mEq/d) [13]. The chronic, low grade metabolic acidosis induced by the modern, western diet is exacerbated during ageing when renal function begins to decline [14,15] requiring the body’s skeletal reserves to be called upon to relinquish bicarbonate to produce alkaline buffers needed to continuously balance the acid load. This results in bone mass that is worn away gradually and indefinitely after the age of 30 years, accelerating at menopause to lower bone strength and mineral density [14,16-18]. F/V’s influence on acid–base balance is crucial as the sole dietary source of alkaline precursor constituents and is an important reason to recommend increased consumption during ageing to forestall bone loss [19,20].

Additional benefits on bone metabolism ensue from bioactive constituents found predominantly in vegetables but also some herbs and fruit. Phytochemicals, antioxidants and other bioactive compounds influence bone metabolism through a variety of mechanisms [21-25] particularly in reducing inflammation and oxidative stress [26,27]. This pharmacological effect on bone resorption was first observed a decade ago by Muhlbauer [28,29] who, in precise and controlled conditions with animals, demonstrated specific vegetables, herbs and fruit positively affected bone resorption quite apart from effects on diet acid load. Muhlbauer determined the effect was additive, therefore, the more of this range consumed, the more bone resorption reduced. This effect has previously been shown only in the animal model.

Intervention studies with mid life women assessing acid load and bone health have been limited to modest increases in self selected fruit and vegetables [8,30], use of supplements [8,31] or use of alkaline water [32,33], mimicking F/V alkali forming effect. No study has increased F/V intake to significantly affect NEAP or specified daily intake of vegetables, herbs and fruit shown in the animal model to have bone resorption inhibiting properties. A diet high in F/V and including some from this range of vegetables, herbs and fruit could be a useful dietary strategy to ameliorate bone loss particularly at critical times such as menopause.

Despite the numerous reports in the literature attributing health benefits with increased consumption of F/V and improvement in chronic disease risk factors [22] most New Zealanders don’t reach the Ministry of Health (M.o.H.) target of 2 servings of fruit and 3 servings of vegetables every day [34,35].

It is hypothesised that an increase in vegetable and fruit consumption to ≥ 9 servings/day will reduce the estimated Net Endogenous Acid Production (NEAP) by approximately 20 mEq/day and result in reduction in bone markers of resorption C telopeptide of type 1 collagen (CTx) and bone formation marker Procollagen 1 N-terminal peptide (P1NP) in post menopausal women, and those women who include 4–5 servings of vegetables, herbs and fruit with bone resorption inhibitory properties (BRIPs) as half of the 9 servings/day will reduce resorption marker CTx by a greater amount. It is also hypothesised that this increase in fruit and vegetable intake will significantly affect inflammatory and metabolic markers including: c-reactive protein (CRP), adiponectin, interleukin 6 (IL-6), interleukin 10 (IL10), tumour necrosis factor (TNF), triglycerides, cholesterol, fibrinogen and plasminogen activator inhibitor-1 (PAI-1). This study therefore aims to investigate the effect of increased fruit, vegetables and herbs on bone, metabolic and inflammatory markers and whether including specific fruit, vegetables and herbs with BRIPs [28] as part of an increased fruit/vegetable intake has any additional effect.

## Methods/Design

**Figure 1:**
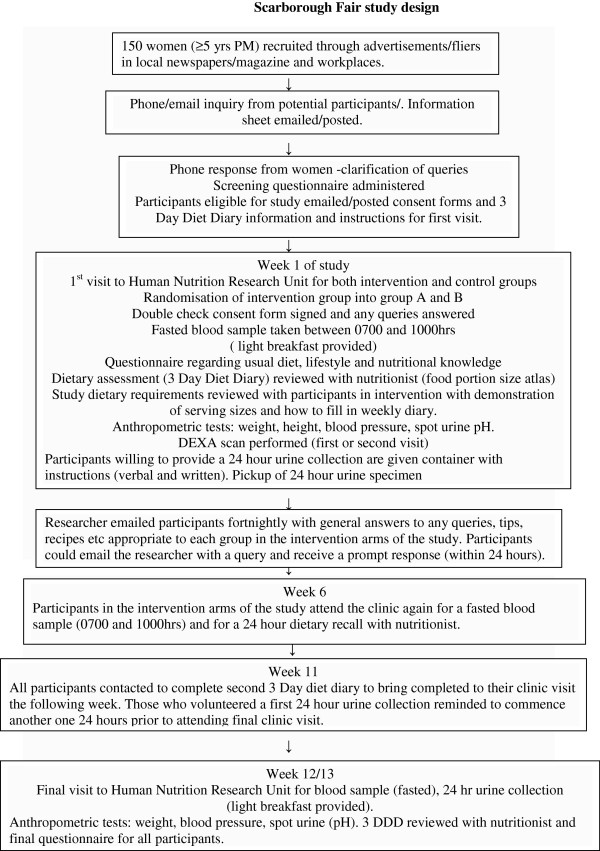
Scarborough Fair study design.

Figure 1 illustrates the study design. This study is a randomized active comparator controlled intervention to increase fruit and vegetable intake in healthy postmenopausal women over a 3 month period.

### Sample size

The number of subjects required in each group was calculated to be 32 (minimum). This was determined using a power calculation based on demonstrating a difference of ~8% in the primary outcome variable C-telopeptides of collagen (CTx) with 80% power and alpha of 0.05 (2 sided test) and accepting 0.4μg/ml as mean CTx of this population (26). To detect any differences between the 2 diets and allowing for withdrawals, non-compliance or maintenance (~ 25%) approximately 50 women were needed in each group. Since there were 2 different diets emphasizing different vegetables and fruit and a control group who consumed their usual diet (≤ 5 servings F/V/day), three groups of 50 participants were required.

### Inclusion/Exclusion criteria

The target population were healthy, post menopausal (≥ 5yrs) women between 50–70 years. Women were included if they were taking some medications e.g. hypertensive tablets, thyroxine (if thyroid function stable) and diuretics other than potassium sparing but excluded if on medication for diabetes, heart disease, osteoporosis (including hormone replacement therapy) or medication that could affect bone or calcium metabolism (oral corticosteroids, warfarin, dilantin. potassium sparing diuretics and regular use of proton pump inhibitors). Regular use of NSAIDs including aspirin was not permitted as they could interfere with anti-inflammatory markers. If participants had stopped use of a NSAID 1 month prior to study commencing they were included. Women were also excluded if they had any of the following conditions: osteoporosis previously diagnosed, both hips replaced, previous fractures of the lower vertebra or hip, severe osteoarthritis* of the lower spine or hips, gastrointestinal, liver or renal disease and any severe* disease including treatment for cancer within the last 3 years. Women who smoked, drank more than 20 standard drinks/week or were already consuming > 6 servings fruit and vegetables every day, or were taking calcium supplements and unwilling to stop a month before the study for the duration of the study were excluded. Any participant who developed an illness during the study that required treatment with steroids or medication that affected bone, inflammatory and other metabolic markers was also excluded. The intervention group participants had to be willing to increase their intake of fruit and vegetables to 9 servings/day and the negative control group willing to continue their normal diet.

*Severe defined as requiring daily pain relief.

### Setting and recruitment

This was a multi-centre trial, with 50 participants at each trial site in Hawke’s Bay, Palmerston North and Auckland. The study was conducted at Massey University’s clinical nutrition research units in Palmerston North and in Albany, Auckland. Hawke Bay participants attended Choices medical centre in Hastings. Participants were recruited using 2 different fliers. One flier recruited 100 women to form the intervention group and be randomised to one of two groups (A or B) within the intervention to increase intake of fruit and vegetables to 9 servings/day. The other flier recruited 50 women (Group C) who were willing to have their bone, inflammatory and metabolic markers tested on two occasions 3 months apart ( baseline and end of study) and who would continue eating their usual diet. This negative control group was called the Diet and Metabolic Markers group (DMM) and referred to in this protocol as Group C. Because of the motivation and commitment involved, it was considered preferable to recruit a negative control group of women separately rather than randomising women to a control group when they were attracted to the study because of a conscious decision to participate in the dietary change. The same exclusion and inclusion criteria applied to the control group apart from the requirement for dietary change.

The study was advertised in local newspapers in the 3 centres, in a few workplaces and in a small advertisement on the health page of The Listener (a popular national magazine) over July/August 2011.This advertising and word of mouth returned a good response rate (> 350 enquiries) with enquiries mainly to participate in the dietary intervention rather than the negative control study (Group C). Recruitment was completed within 6 weeks of first advertising.

### Screening

Prospective participants who phoned or emailed expressing an interest in the study were initially sent out the appropriate detailed information sheet for perusal. If they replied (email/ phone) willing to participate, a screening questionnaire was completed over the phone. This questionnaire included demographics, health status (including medications) and biographic information. Over 300 women were screened with over half being declined due to a significant health issue or on medication deemed incompatible with the study e.g. regular use of proton pump inhibitors.

### Randomisation and blinding

Participants were stratified according to the 3 cities and randomly allocated to either group (A or B) using block randomization. The random allocation sequence was generated by administrative personnel (not the researcher who recruited participants) and intervention group participants were assigned to group A or B as they arrived for their first appointment.

As intervention participants were required to source, store, prepare and consume specific vegetables, fruits and herbs they were not blinded to which diet (A, or B) they were on once randomisation had occurred, but were blinded to which group contained the fruit, vegetables and herbs with BRIPs. The randomization codes were maintained by an alpha numeric added to the participant’s unique identifier and laboratory staff involved in the analysis of blood or urine samples were blinded to participants’ group.

### The intervention rationale

Women in the intervention groups A and B were asked to consume a minimum of 9 servings a day of fruit and vegetables with herbs additional. 9 servings per day was chosen as the target number of fruit and vegetable servings as it allows for a significant decrease in estimated net endogenous acid production (NEAP) of the diet (~20 mEq/day unpublished feasibility study) and was the number of servings recommended by the USDA for healthy, fit 50 year old women [36] as well the number recommended in the DASH Trial [37]. This increase in fruit and vegetables could then be compared to a group of 50 women consuming their normal diet (≤5 servings/day).

In order to assess the impact of BRIPs both groups were asked to emphasise a different selection of vegetables, herbs and fruit and avoid others. Group B include 4–5 servings of specific vegetables, fruit and herbs with bone resorption inhibitory properties (BRIPs), while Group A avoided those specific vegetables, herbs and fruit and emphasised other specific fruit, vegetables and herbs. To allow for change in bone turnover markers particularly P1NP, the minimum study period was determined to be 3 months [38].

Bone resorption inhibiting properties have been ascribed to a limited range of common fruit, vegetables and herbs. These include the herbs dill, sage, garlic, parsley, thyme and rosemary. Vegetables and fruit with BRIPs include tomatoes, green beans, cucumber, broccoli, lettuce, prunes and oranges [28]. The minimum effective dosage of fruit/vegetables and herbs (F/V/H) with BRIPs calculated in the animal model is 170 mg/day. This corresponds with 6.2 grams of fresh F/V/H per kilogram human body weight. This amount is equivalent to 3–5 servings /day [35] of any F/V with BRIPs for a 60–70 kilo woman. Culinary herb servings were to be additional and usual culinary measures in meals were advised to all intervention participants (2–3 cloves garlic, 0.5-1 teaspoon for dried/fresh culinary herbs and up to 0.25 cup of parsley (Group B-BRIPS) and basil (Group A- non-BRIPS). The effect on bone resorption of the foods with BRIPs is said to be additive [39]. Vitamin K is also known to affect bone health and fracture risk through its action on cytokines and the gamma carboxylation of the bone protein osteocalcin [40-42]. To control for vitamin K intake all participants in the intervention were asked to include one serving of a leafy green vegetable in their diet every day. They were also asked to have at least 2 servings of dairy or calcium enriched soy milk or other alternative to control for calcium intake [35]. Twice a week the intervention group women recorded their total vegetable, fruit and herb intake as well as their urine pH (fasting, second void) to the nearest 0.25 ph unit with dipsticks provided (PHion Diagnostic Test Strips, Apex Wellness Group, UC, Scottsdale, AZ85260, USA).

***Group A*** ≥ 9 servings of fruit and vegetables (≤ 3 servings fruit and ≥ 6 servings of vegetables) Specific fruit/ vegetables/herbs (all non BRIPs) specified for over half the servings and avoiding F/V/H with BRIPs.

***Group B*** ≥ 9 servings of fruit and vegetables (≤ 3 servings fruit and ≥ 6 servings of vegetables) Specific fruit/ vegetables/herbs (all BRIPs) specified for over half the servings and avoiding some of Group A’s specified fruit/vegetables/herbs.

***Group C*** Negative control group who consume their usual diet

### Blood and urine sampling

Procedures for taking blood, urine and anthropometric measurements were standardized to reduce errors and variability. Bone markers show circadian variability [43] therefore all fasted blood samples were taken between 0700 and 1000hr with blood drawn by certified phlebotomists. After their first appointment women were advised to make their subsequent appointments as close as possible to the exact time of day as their first and most were able to comply with this. Plasma was used for analysis of bone, inflammatory and metabolic markers and drawn into vacutainers containing EDTA, citrate or heparin. After centrifugation at 3000 rpm for 15 minutes (4°C), the plasma was dispensed in aliquots and frozen at −80°C. Participants collected a 24 hour urine sample the day prior to attending their clinic visits. At the clinic the sample quantity was measured and 3 aliquots of 100 ml were frozen immediately at −20°C. All blood and urine samples were analysed at the end of the 3 month study period. To reduce inter-assay variability both baseline and end of study samples were analysed in the same assay run.

### Biochemical analysis

Refer Table 1 for details of biochemical analysis done.

**Table 1:** Outcome variables and analysis method

**Outcome Variables**	**Baseline**	**Mid 6 wks**	**End** (**3mths**)	**Method**
**CTx bone resorption marker**	I & C	I	I & C	Roche Elecsys 2010.Roche Diagnostics, Canterbury Health Endocrine laboratory (CHL). Accredited with IANZ to the ISO 15189
**P1NP bone formation marker**	I & C	I	I & C	Roche Elecsys 2010.Roche Diagnostics, Canterbury Health Endocrine laboratory.
**Cholesterol**	I & C		I & C	CHL Enzymatic Cholesterol Oxidase Abbott c8000 analyser. Abbott reagents
**Triglycerides**	I & C		I & C	CHL Enzymatic hydrolysis of Triglycerides. Abbott c8000/c16000 analyser, Abbott reagents.
**LDL**	I & C		I & C	CHL calculated as follows: LDL Chol = Total Chol - HDL Chol -(Trigs/2.22) mmol/L.
**HDL**	I & C		I & C	CHL Enzymatic assay.Abbott c8000 analyser.Abbott reagents
**Fibrinogen**	I & C		I & C	Fibrinolysis reference plasma (Cat. No. T6010) (Kordia, Leiden, The Netherlands).
**PA1**	I & C		I & C	Plasma PAI-1_act_ bio-immunoassay, TriniLIZE PAI-1 activity assay kit (Cat. No. T6004)
**CRP**	I & C		I & C	Plant and Food Research Auckland FlowCytomix kits (Bender Medsystems, Austria) FC500 MPL flow cytometer (Beckman Coulter, Miami, FL
**IL6**	I & C		I & C	As above
**IL10**	I & C		I & C	‘’
**TNF alpha**	I & C		I & C	‘’
**OPG**	I & C		I & C	‘’
**Adiponectin**	I & C		I & C	‘’
**24hr Calcium**	I & C		I & C	E.I.T Research Laboratory - Shimadzu AA-6200 Atomic Absorption Flame Emission Spectrophotometer with air/acetylene flame.(Method adapted from Gimblet El)
**24hr Magnesium**	I & C		I & C	AAS as above (Method adapted from Gimblet El)
**24hr Sodium**	I & C		I & C	AAS as above (Method adapted from Castenmillar JM)
**24hr Potassium**	I & C		I & C	AAS as above (Method adapted from Castenmillar JM)
**24hr Creatinine**	I & C		I & C	Massey University Nutrition laboratory. Jaffe Method Flexor E, Vital Scientific NV, 6956 AV Spankeren/Dieren, The Netherlands
**Iodine**	I & C		I & C	Hills Laboratory - inductively-coupled plasma mass spectrometry.
**Selenium**	I & C		I & C	Hills Laboratory - inductively-coupled plasma mass spectrometry
**Dietary assessment**				
**3DDD macro and micronutrient intake**	I & C		I & C	3 day food intake (2 week days and 1 weekend) Recipes/packaging.
				Foodworks NZ,2009 (Xyris software)
**24 hour dietary recall**		I		Previous day 24 hour recall second clinic visit nutritionist (NZ reg.)
***Estimated *NEAP and PRAL**	I & C		I & C	Derived from algorithms ([44]) and using Food works (Xyris, NZ) data from 3DDDs
**No**. **serves each food groups**	I & C		I & C	Derived from 3DDD nutritionist (NZ reg. ) converted quantities in grams/mls/cups etc. to servings ([35]
**F**/**V**/**Herb Diary**	Intervention group only. Twice weekly- self reported intake			
**Urine ph** (**2**/**wk**)	Intervention group Twice weekly self reported urine pH (second void /fasting) Phion Diagnostic Test Strips (0.25 graduations), Apex Wellness Group, UC, Scottsdale, AZ85260, USA			
**Weight**/**height BMI**	I & C		I & C	Digital scales UWE Gilbarco, NZ / Stadiometer/ BMI =weight/height^2^
**Blood pressure**	I & C		I & C	Omron Digital HEM-907 automatic blood pressure monitor (duplicates) high values repeated manually
**Waist**/**hip measurements**	I & C		I & C	Anthropometry methods performed by trained nutrition staff using standardised procedures and equipment. Tape measure, Douglas Pharmaceuticals Limited, Auckland.
**Body composition and BMD**	I & C	DEXA Hologic Discovery **QDR 4500A** densitometer, Hologic Inc. Bedford, Massachusetts		

Intervention (I) and control group (C).

### Dietary assessment

3 Day Diet Diaries (3DDDs) were done at baseline and end of study (week 12). Participants received written and verbal instruction on how to complete the 3DDD’s via email and phone call prior to attending their first clinic appointment. They were asked to record all food and beverages consumed over 2 weekdays and 1 weekend day, including types, brands and amounts (cups, tablespoons, etc.) of foods as well as recipes for homemade dishes. Participants supplied nutritional information panels from processed food packets. All 3DDD’s were checked for accuracy and completeness by a NZ registered nutritionist at the first visit. Prompting methods were used for incomplete quantities or to ascertain specific food types e.g. milk (skim or full fat).

A 24 hour recall (unexpected) was done with intervention participant’s midway through the study at the second clinic visit. Dietary data were entered into Foodworks (version 2009, NZ, Xyris software) by a M.Sc (nutrition) graduate, checked for accuracy and completeness by a N.Z. registered nutritionist and then transferred to SPSS (20) via Microsoft Access (2007) and Excel (2007).

New Zealand dietary reference values were used to assess all nutrient intakes [35]. Net endogenous acid production (NEAP) was calculated according to the algorithm from Remer and Manz [44,45].

NEAP = Potential Renal Acid Load (PRAL) + Organic Acid (OA)

PRAL represents the average intestinal absorption rates of ingested protein and additional minerals. OA is an anthropometry-based estimate for organic acid excretion.

The two components of NEAP are given as follows:

PRAL (mEq/d) = 0.49 protein (g/d) + 0.037 × phosphorous (mg/d) - 0.021 × potassium (mg/d) – 0.026 × magnesium (mg/day) - 0.013 × calcium (mg/day)

OAest (mEq/day) = individual body surface area × 41/1.73

Body surface area was calculated according to the method of du Bois and du Bois

BSA = (W ^0.425^ × H ^0.725^) × 0.007184

(Website http://http:/www-users.med.cornell.edu/~spon/picu/calc/bsacalc.htm)

NEAP and PRAL are expressed in milliequivalents (mEq) as ions in solution interact according to their charge. Milliequivalents is obtained by converting milligrams (mg) to millimoles (mmol) and multiplying by valences to give charge quantities [44]

### Questionnaires

At the first clinic visit a baseline questionnaire was administered with the intent of focusing participants on their motivations for this behaviour change and how they would overcome common barriers to dietary change [46] and how they would incorporate the vegetables, fruit and herb increase required into their daily dietary intake. The final study questionnaire assessed the participant’s experience of the dietary change, both positive and negative aspects, including financial costs and lifestyle changes they made.

### Compliance diaries

Intervention participants were provided with a weekly diary in which they recorded their intake of fruit, vegetables, herbs (type and quantity). and urine pH (fasted second void) on 2 days each week for the duration of the study. This diary was to aid compliance with the dietary requirements and also to see if urine pH increased with increased amounts of fruit and vegetables [47,48]. An increase of 0.68 pH units was previously demonstrated (unpublished feasibility study, 2010) and was said by some participants to be a motivator.

### Adverse event reporting

Participants were advised to report any side effects from the increase in vegetables and fruit intake. They also recorded in their diaries any incidence of adverse side effects e.g. stomach upset, which was reviewed at each clinic visit. The study’s clinical collaborator was available to provide guidance on medical issues. Participants were advised to report immediately their concerns with any serious adverse side effect from this dietary regimen e.g. chronic diarrhoea (> 48 hours). Any participant recording serious adverse effects due to the diet would have been immediately advised to consult their medical practitioner for assessment if they had not already done so and withdrawn from the study.

Follow-up: The researcher/clinical collaborator would follow-up with any participant that had a medical issue, that they had received medical treatment and the issue was resolved satisfactorily. In the unlikely event of a serious adverse side effect from participation in this intervention study, the individual after consultation with their medical practitioner would have been advised to contact ACC (Accident Compensation Corporation). No significant adverse side effects however, were reported.

### Statistical methods

SPSS 20 and Minitab 16 will be used. Repeated measures and one way analysis of variance (ANOVA) will be done on serum bone markers (baseline, 6 and 12 weeks) to determine change over the study period, multiple regression and Pearson’s correlation will also be used for daily F/V intakes and bone markers. Analysis of nutrient intake before and during the intervention will be by paired *t*-tests and repeated measures, Main outcome variables will be checked for normal distribution (Shapiro-Wilk test) and any data not normally distributed will be log transformed before comparisons are made. A level of significance of 0.05 will be used

The primary analysis will be the comparison of change in bone markers between the intervention groups and negative control group following the “intention to treat” principle at 6 weeks and 3 months. Secondary analyses will involve any intervention treatment effects on inflammatory and metabolic markers. Per protocol analyses will also be performed and compared with intention to treat to assess bias.

### Participant’s results

All participants received individualised dietary assessment analysis and their DEXA body composition results but not individual blood biochemistry or urinary test results (requirement of MUHEC).

#### Dietary analysis

Following input of 3DDD’s into Foodworks (2009), all participants in group C (negative control group) received an overview of their baseline three day diet diary analysis. This provided detailed information on macronutrient and micronutrient intake. Due to this study involving dietary change, all intervention participants received both beginning and ending 3DDD results to assess micronutrient changes associated with their increased vegetable and fruit intake. Participants with queries about their nutrient analysis were invited to email the nutritionist (NZ reg.) and received immediate feedback.

#### DEXA analysis

All participants received a letter informing them of their DEXA bone scan result. This was reported as normal (including mild osteopenia), osteopenia or having a T score ≥ 2.5 SDs below normal. Participants with DEXA results not within normal parameters for their age group (latter 2 groups) also received the radiologists report on their bone scan with their DEXA t and z scores details and a letter advising them to discuss with their doctor.

### Funding and ethics

Funding for this study was provided from the following organisations: Hawke’s Bay Medical Research Foundation, The National Heart Foundation, Massey University Research Fund (MURF), and Amgen/GSK Clinical Grants Program administered by the OA-ANZBMS Research Fund.

This study was approved by Massey University Human Ethics Committee (MUHEC) (Southern A) Reference 11/11 in June 2011. It was conducted in compliance with the protocol approved by the Massey University Ethics Committee (MUHEC) and will be reported according to CONSORT guidelines. No deviation from the protocol was implemented without the prior review and approval of MUHEC. Participants were informed they could withdraw from the study at any point. All subjects for this study were provided with a detailed information sheet describing the study and providing sufficient information to make an informed decision about participation before they gave signed consent.

## Discussion

This study “The Scarborough Fair study” will provide detailed information about the influence of increased fruit and vegetable intake on the bone health of middle aged New Zealand women (50 -70years). The intervention involves a significant behaviour change and this increase in fruit, vegetables and herbs may be expected to have an effect on inflammatory, metabolic and bone measures of health. The strengths of the study are that it includes a combination of prescribed fruit, vegetables and herbs previously shown to affect bone resorption in the animal model but not previously trialled in humans [39], its primary emphasis on vegetables (≥ 6 servings) rather than fruit (≤ 3 servings) [49] and the inclusion of herbs [24]. This differs from previous intervention studies on bone health using the fruit and vegetable food group [8,30]. Limitations with the study include the element of selection bias introduced as a result of stringent inclusion and exclusion criteria. In some ethnic groups, health status is likely to be already compromised by midlife due to chronic illness (diabetes and heart disease) and these groups were under represented. Predominantly European women formed the study population. A high element of commitment to a behaviour/lifestyle change was required with the need to purchase, store and prepare all fruit and vegetables and herbs consumed. There were no financial incentives and participants received a petrol voucher ($10/per visit) at conclusion of the study.

Results from this study may provide further evidence to encourage increased consumption of fruit, vegetables and herbs with emphasis on those with bone resorption inhibiting properties.

## Abbreviations

ACC: Accident Compensation Corporation; AI: Adequate intake; BRIPs: Bone resorption inhibiting properties; BSA: Body surface area; CRP: C-reactive protein; CTx: C teleopeptide (cross linked C terminal of α chain of type 1 collagen); DEXA: Dual energy X ray absorptiometry; EAR: Estimated average requirement; F/V: Fruit and vegetables; HRT: Hormone replacement therapy; IL-6: Interleukin 6; IL-10: Interleukin 10; MUHEC: Massey University Human Ethics Committee; MoH: Ministry of Health; NEAP: Net endogenous acid production; NSAIDs: Non steroidal anti-inflammatory drugs; NF κB: Nuclear factor kappa binding; OA: Organic acid; PAI-1: Plasminogen activator inhibitor-1; P1NP: Procollagen 1 N-terminal peptide; PRAL: Potential renal acid load; RDI: Recommended daily intake; SDT: Suggested dietary target; TNF: Tumour necrosis factor; 3DDD: Three day diet diary; USDA: United States Department of Agriculture.

## Competing interests

The authors declare no competing interests.

## Authors’ contributions

CG designed the study with help from JW and MCK. CG drafted the manuscript. JW and MCK provided comments on and accepted the final version of the manuscript. All authors read and approved the final manuscript.

## Authors’ information

CG is a registered nutritionist completing a PhD in Nutritional Science at Massey University. JW is a senior lecturer in nutrition at Massey University. MK is Professor and Chair of Nutritional Physiology. MK has research expertise in human nutrition and health with a major emphasis on bone health and specialist expertise in in vitro assessment of bioactives, and conducting nutritional trials in human and animal models.

## Pre-publication history

The pre-publication history for this paper can be accessed here:

http://www.biomedcentral.com/1471-2458/13/23/prepub
